# A blood-based immune-suppressive index stratifies immunotherapy outcomes in advanced NSCLC

**DOI:** 10.3389/fimmu.2026.1772319

**Published:** 2026-05-08

**Authors:** Luca Lalli, Veronica Huber, Agata Cova, Elena Daveri, Angela Listì, Giovanni Rossi, Carlo Genova, Simona Coco, Iosune Baraibar, Ignacio Gil-Bazo, Silvia Novello, Licia Rivoltini, Francesco Passiglia

**Affiliations:** 1 Translational Immunology Unit, Department of Experimental Oncology, Fondazione IRCCS Istituto Nazionale dei Tumori, Milan, Italy; 2 Department of Oncology, University of Turin, S. Luigi Gonzaga Hospital-Orbassano, Turin, Italy; 3 UOC Oncologia Medica, IRCCS Azienda Ospedaliera Metropolitana (IRCCS AOM), Plesso: Ospedale Policlinico San Martino, Genoa, Italy; 4 Dipartimento Di Medicina Interna E Specialità Mediche (DiMI), Università Degli Studi Di Genova, Genoa, Italy; 5 Department of Oncology, Clínica Universidad de Navarra, Pamplona, Spain; 6 Program in Solid Tumors, Center for Applied Medical Research and Navarra Institute for Health Research, Pamplona, Spain

**Keywords:** biomarker, cytokines, immunotherapy, liquid biopsy, neutrophils, NSCLC, PD-L1

## Abstract

**Background:**

Immune checkpoint inhibitors (ICIs) provide durable benefit for a subset of patients with advanced non-small cell lung cancer (NSCLC), however predictive biomarkers remain limited. Peripheral blood offers accessible immune and inflammatory signals that may reflect the onset of systemic mechanisms of resistance. This study aimed to identify circulating immune features associated with clinical outcome and to develop a clinically applicable blood-based score for risk stratification in patients receiving ICIs.

**Methods:**

Two independent real-world cohorts of advanced NSCLC patients treated with anti-PD-1 therapy were analyzed. Plasma cytokines and chemokines, soluble immune checkpoint molecules and blood cell counts were measured before and during ICI-therapy. Baseline biomarkers associated with progression-free survival (PFS) and overall survival (OS) were incorporated into an Immune-Suppressive Blood Index Score (ISBIS). The ISBIS discriminative performance was first determined in the discovery cohort (n=57) and then validated in an independent cohort (n=56), and nomogram integrating ISBIS with clinical variables was generated.

**Results:**

ISBIS score predicts poor outcome to be associated with an immunosuppressive blood profile composed of baseline IL-6, IL-8, CCL2, CXCL10, neutrophils, PLR, IFN-γ and lymphocytes. On-treatment increase in CXCL10, neutrophils, and NLR, as well as decreases in lymphocytes and soluble PD-L2, were associated with early disease progression. ISBIS stratified patients into three risk groups with significantly different survival in both cohorts and remained independently associated to OS. A nomogram combining ISBIS with ECOG-PS (Eastern Cooperative Oncology Group - Performance Status), disease burden, sex, and NLR (neutrophil-to-lymphocyte ratio) demonstrated strong discrimination and validated accuracy.

**Conclusions:**

A systemic immune-suppressive profile identifies patients with poor outcomes to ICIs. ISBIS integrates critical immune and inflammatory signals into a clinically applicable tool, supporting personalized immunotherapy strategies in advanced NSCLC.

## Introduction

The introduction of immune-checkpoint inhibitors (ICIs) targeting the programmed death-1 (PD-1)/programmed death ligand-1 (PD-L1) axis has profoundly transformed the therapeutic landscape of advanced non-small cell lung cancer (NSCLC). Over the last decade, anti-PD-1/PD-L1 agents have demonstrated the ability to induce deep, durable clinical responses in a subset of patients, with marked improvements in overall survival (OS) and quality of life (QoL) (1). Following the initial approval of nivolumab, pembrolizumab, and atezolizumab for previously treated, NSCLC patients (2–4), ICIs have rapidly expanded to first-line settings. Today, PD-1/PD-L1 inhibitors are standard of care as monotherapy for tumors with high PD-L1 expression (1) and in combination with platinum-based chemotherapy regardless of PD-L1 status (5–7). Long-term follow-up from pivotal trials has confirmed that ICIs have changed the natural history of metastatic NSCLC, with five-year OS rates reaching approximately 15% in the second-line (2) and 30% in the first-line setting for PD-L1 high tumors (1) compared with ~5% in the pre-immunotherapy era. This advance highlights for the first time the possibility of achieving long-term disease control and potentially functional cure in selected patients with metastatic disease. However, only a minority of patients experience durable benefit from ICIs, while tumor heterogeneity, host immune variability, and both primary and acquired resistance mechanisms significantly limit treatment efficacy (8). Consequently, the identification of reliable biomarkers capable of predicting immunotherapy response has become a critical unmet need. To date, several tissue-based biomarkers, such as tumor PD-L1 expression, tumor mutational burden (TMB), tumor-infiltrating lymphocytes (TILs) and gene-expression signatures have been evaluated (8). However, their predictive accuracy is modest, their distribution frequently overlaps between responders and non-responders, and their assessment is limited by tumor sampling constraints and spatial/temporal heterogeneity. Conversely, peripheral blood-derived biomarkers could provide a more reliable and readily quantifiable easy to assess picture of antitumor immune-response, offering an attractive alternative for clinical use. Indeed, circulating immune cells, cytokines, chemokines and soluble factors, including soluble immune-checkpoint molecules, constitute a dynamic and accessible source of information that reflects the systemic immunological state, tumor-host interactions and evolving mechanisms of resistance (9). Increasing evidence suggests that blood-based immune signatures may capture clinically meaningful patterns of inflammation, immune activation and immune suppression, which influence therapeutic response (8).

In this study, we investigated the relationships between plasma cytokine-chemokine profiles, soluble immune-checkpoint molecules and peripheral routinely counted immune cell populations in two independent real-world cohorts of patients with advanced NSCLC treated with anti-PD-1 therapy. Through integrative analysis, we identified a coordinated set of circulating immune features associated with systemic immunosuppression and poor clinical outcome. From these components, we developed the Immune-Suppressive Blood Index Score (ISBIS) and validated its discriminative performance across cohorts.

## Materials and methods

### Study population

A total of 113 eligible patients with previously treated advanced NSCLC who received nivolumab in the second-third line setting, with available pretreatment and/or on-treatment plasma samples, were included in this retrospective study and grouped as follows: Cohort A (study group): 57 advanced NSCLC patients enrolled at S. Luigi Hospital (Orbassano (TO), Italy); Cohort B (validation group): 56 advanced NSCLC patients enrolled at S. Martino Hospital (Genova, Italy). All the patients participated in the PROMOLE translational study (ethics approval number 73/2018 of 2024.01.30) and signed and dated an informed consent and privacy form. Upon signing such a specific informed consent and privacy forms, patients indicated that they understood the purpose of and procedures required for the study and were willing to participate in the study, allowing the collection of both biologic samples and source data verification in accordance with Italian requirements, if applicable. We retrospectively collected clinical and pathological data as well as routine blood tests from patients’ charts and electronic medical records. Patients included in the analysis received ≥1 dose of anti-PD1 inhibitors. The treatment was continued until disease progression or unacceptable toxicity. Radiological evaluation of treatment efficacy by CT-scan was performed every 12 weeks until disease progression and responses were evaluated by Response Evaluation Criteria in Solid Tumors (RECIST) v1.1. The study was conducted in accordance with the International Conference on Harmonization Guidelines on GoodClinicalPractice (GCP) and the Declaration of Helsinki.

### Peripheral blood collection and analysis

Routine blood examination was obtained within one week prior to first and fourth (8^th^ week) administration of PD-1/PD-L1 inhibitor and included the white blood cell count (both, lymphocytes and neutrophils) and the platelets count, from which the neutrophil-to-lymphocyte ratio (NLR) and the platelet-to-lymphocyte ratio (PLR) were deduced.

Pre-treatment and/or on-treatment peripheral blood samples were collected at two different Italian Institutions. In detail, pretreatment blood was drawn on the same day prior to the first administration of PD-1/PD-L1 inhibitor for 113 patients (57 patients from cohort A and 56 patients from cohort B). On-treatment peripheral blood samples were collected on the same day prior to the fourth administration (8^th^ week) of PD-1 inhibitor for 37 out of 57 patients enrolled in the cohort A and for 56 out of 56 patients enrolled in the cohort B. Plasma was obtained from whole blood EDTA samples. Overall, two aliquots (2.0 ml) of plasma from each time point were isolated after two consecutive centrifugation steps (2300 revolutions per minute for 10 minutes) and stored at −80 °C until further use.

Cytokine Bead Array (CBA, Becton Dickinson) allows the flow cytometry-based quantification of multiple proteins simultaneously via antibody-coated beads. According to manufacturer’s instructions, standard curves were run in duplicate to ensure accuracy, while the assay was run with single replicates for experimental samples due to its high precision and limited sample requirements. Plasma samples (50 µl) from cohort A were analyzed for the presence of the cyto-chemokines IL-6, IL-8, TNFα, CCL2, CX3CL1, Granzyme B, IFN-γ, CXCL10, CD62L, CCL2, VEGF-A by CBA according to manufacturer’s instructions. Samples were acquired with a FACSCalibur flow cytometer (Becton Dickinson), and data were analyzed by the FCAP Array software (Becton Dickinson) software. Soluble immune checkpoints, comprising TIM3, CD28, CD137, CD27, CD152, HVEM, IDO, LAG3, BTLA, GITR, CD80, PD-1, PD-L1, PD-L2, were quantified in plasma samples of the 37 paired samples (pre-post) of cohort A by multiplex cytokine assay (Luminex) following manufacturer’s instructions, with all samples run in duplicate.

Validation of the cytokines composing the ISBIS score (IFN-γ, IL-6, IL-8, IP10 CCL2) was then performed by CBA on plasma samples of cohort B according to the experimental conditions used for cohort A analyses, as described above.

### Statistical analysis

Standard descriptive statistics (absolute and relative frequencies for categorical variables, mean, medians, standard deviation and interquartile (IQ) ranges for continuous variables) were used to describe the sample pre-post and delta characteristics. The Mann Whitney test was used for intergroup comparisons of two independent samples while X^2^ or Fisher’s exact test was used for categorical values, as appropriate. Correlation analyses were done using non-parametric Spearman’s rho coefficient to investigate pairwise associations among the biomarkers and clinical variables.

Overall survival and progression free survival were calculated as the intervals between the date of treatment start and the date of death for any cause/relapse, with censoring occurring at the date of the last follow up visit for event-free patients. These endpoints were described by Kaplan-Meier curves and analyzed with univariable Cox regression models.

Multiple imputations were performed 1000 times to ensure stability of the mean imputed value for each missing value. The machine learning method AIM (Adaptive Index Modeling) was used to create multivariate Cox regression models after dichotomizing the variables. The method incorporates variable selection by employing binary rules and a forward selection procedure based on statistic scores.

This is a synergistic model where the contribution of each variable, defined by its specific threshold and directionality (> or <), is determined by its ability to optimize the overall index’s discriminative performance. Within this integrated algorithm, a variable may contribute to the high-risk profile in a specific direction because that configuration, when analyzed in combination with the other parameters, provides the most robust stratification of clinical outcomes, independently of isolated univariate trends. Cross-validation was used to determine the final number of variables retained in the model. The output was an index score that identified a subgroup of variables exceeding the identified cut-offs at the individual patient level.

The conventional two-sided 5% level was chosen as the threshold of statistical significance.

All statistical analyses were performed with R software (version 4.4.1, R Foundation for Statistical Computing, Vienna, Austria).

## Results

### Independent real-world cohorts characteristics

Baseline demographic and clinicopathological characteristics of patients included within the cohorts A and B of this study (**Table 1**) revealed: for the cohort A, the discovery cohort, that patients’ median age was 66 years (range 51-80), the majority of patients were males (68.4%), current or former smokers (77.2%) and exhibited an ECOG PS (Eastern Cooperative Oncology Group – Performance Status) <2 (87.7%). The bone was the most common metastatic site (43.9%) followed by liver (33.3%), adrenal gland (26.3%) and central nervous system (CNS) (12.3%). The majority of patients (55.5%) had adenocarcinoma subtype. ICIs were preferentially administered as second-line treatment (63%). Median PFS and OS in the overall cohort A population were 5 months and 12 months, respectively, with 16% of patients experiencing early progression (early PD, progressive disease, by RECIST at the first CT-scan evaluation). Patients’ median follow-up was 20 months (IQR: 11–22).

**Table 1:** Baseline demographic and clinicopathological characteristics of patients in cohort A and cohort B.

Characteristic	Cohort A N = 57^1^	Cohort B N = 56^1^
Gender		
* Male*	39 (68.4%)	33 (58.9%)
* Female*	18 (31.6%)	23 (41.1%)
Smoker		
* No*	13 (22.8%)	7 (12.5%)
* Yes*	44 (77.2%)	49 (87.5%)
Early progression		
* No*	48 (84.2%)	34 (60.7%)
* Yes*	9 (15.8%)	22 (39.3%)
Age > 70		
* No*	42 (73.7%)	27 (48.2%)
* Yes*	15 (26.3%)	29 (51.8%)
Performance status > 2		
* No*	50 (87.7%)	51 (91.1%)
* Yes*	7 (12.3%)	5 (8.9%)
Metastasis		
* M1_a*	17 (29.8%)	22 (39.3%)
* M1_bc*	40 (70.2%)	34 (60.7%)
Squamous		
* No*	41 (71.9%)	45 (80.4%)
* Yes*	16 (28.1%)	11 (19.6%)
Second line		
* No*	21 (36.8%)	8 (14.3%)
* Yes*	36 (63.2%)	48 (85.7%)

^1^n (%)

Data are presented as frequencies and percentages for each variable.

For the cohort B, the validation cohort, the proportion of male patients was slightly lower (58.9%), most of them (87.5%) were current or former smokers, with adenocarcinoma subtype (80.4%) and exhibited an ECOG-PS <2 (91%). A higher proportion of patients aged over 70 was recorded in the cohort B (51.8%) versus cohort A (26.3%), as well as a higher number of early progressors (39%). ICIs were primarily administered as second-line treatment (85.7%). Median PFS and OS in the overall cohort B population were 6 months and 13 months, respectively. Patients’ median follow-up was 32 months (range: 17–34) at the end of data collection.

### Systemic immune factors associated with Immunotherapy effectiveness

Baseline biomarkers levels, including both cytokines and peripheral blood cells, were assessed in both study cohorts (**Table 2**). To investigate peripheral blood component dynamics and identify potential biomarkers associated with ICIs’ effectiveness, we first performed an exploratory analysis of cyto-chemokines and soluble immune checkpoints in pre-treatment plasma samples of 57 NSCLC patients from the cohort A and 56 plasma samples from the cohort B, and then we assessed their association with both PFS and OS. In cohort A, pre-treatment IL-6, IL-8, and CCL2 levels were significantly higher in patients with median OS < 12 months while increased IFN-γ levels were found in patients with median OS > 12 months. In addition, an increased absolute number of leukocytes, neutrophils and NLR were observed in patients with median OS < 12 months (**Supplementary Figure 1A**). Similar results were obtained for cohort B, although we could not record statistically significant differences between patients with median OS < 12 months vs OS > 12 months (**Supplementary Figure 1B**). Of note, systemic IFN-γ levels of patients with median OS > 12 months of cohort A were remarkably higher than in cohort B (**Supplementary Figure 1B**).

**Table 2:** Baseline biomarker levels in cohort A and cohort B.

Characteristic	Cohort A N = 57^1^	Cohort B N = 56^1^
IL-6	6.36 (3.30, 16.42)	9.05 (2.06, 21.59)
IL-8	12.57 (5.75, 23.90)	12.05 (8.59, 22.65)
CCL2	27.62 (15.03, 60.40)	38.89 (22.07, 59.49)
CXCL10	241.44 (137.55, 339.71)	160.68 (120.90, 239.15)
* Missing*	1	1
IFN_gamma	4.52 (0.00, 17.22)	0.36 (0.16, 0.91)
Lymphocytes	1.42 (1.02, 1.87)	1.32 (1.10, 1.58)
* Missing*	0	6
Neutrophils	5.39 (3.98, 6.80)	5.06 (3.20, 6.58)
* Missing*	0	2
Platelets	285.00 (212.00, 344.00)	260.50 (202.00, 329.00)
* Missing*	0	2
NLR	3.39 (2.48, 5.39)	3.30 (2.22, 6.56)
* Missing*	0	6
PLR	196.74 (135.14, 291.37)	190.50 (153.13, 270.77)
* Missing*	0	6

^1^Median (Q1, Q3).

Data are presented as median values with interquartile ranges (Q1, Q3) for each biomarker. Missing values are indicated where applicable. NLR, neutrophil-to-lymphocyte ratio; PLR, platelet-to-lymphocyte ratio.

Next, we performed a longitudinal analysis of cyto-chemokine dynamics in samples of both cohorts after 4 cycles of ICI therapy, including soluble checkpoints for cohort A, and routine blood count-based ratios NLR/PLR. In matched sample pairs of 37 out of 57 patients of cohort A we observed an increase in CXCL10, NLR, together with a decrease in lymphocyte count in patients with median OS < 12 months. In contrast, plasma samples of patients with median OS > 12 months displayed higher IL-8 levels with respect to baseline estimations (**Supplementary Figure 2A**). Interestingly, CXCL10 dynamics in cohort B were complementary to cohort A, showing a marked statistically significant increase in the on-therapy sample in patients with median OS < 12 months, while a decrease of IL-8 after 4 cycles of ICI therapy was observed in patients with median OS > 12 months (**Supplementary Figures 2A, B**). Furthermore, soluble immune checkpoint quantification in cohort A revealed a contemporaneous decrease of soluble PD-L2 in the on-therapy sample vs baseline in plasma of patients with median OS < 12 months (**Supplementary Figure 3**), while this data was not available for the cohort B.

A small number of patients in both cohorts (cohort A n=9/57, cohort B n=22/56) experienced early PD, defined as PD < 3 months under ICI-therapies and we deemed particularly relevant to verify cyto/chemokine and soluble checkpoint dynamics in this group. At baseline we could not record significant differences between patients experiencing early PD vs non-early PD in any of the cohorts (**Supplementary Figures 4A, B**). However, longitudinal analysis showed a significant increase between on-treatment versus pre-treatment plasma levels of CXCL10 and IL-8, which was particularly evident in early PD patients of both cohorts, together with significantly increased peripheral blood counts of neutrophils and NLR in early PD patients of cohort A, while sPD-L2 soluble immune checkpoint trended to stronger decrease in early PD patients. These results confirmed these characteristics as especially indicative of worse outcome to therapy with ICI in this clinical setting (**Supplementary Figures 5**, **6**).

### Immune-suppressive blood index score stratifies Immunotherapy response

**Figure 1:**
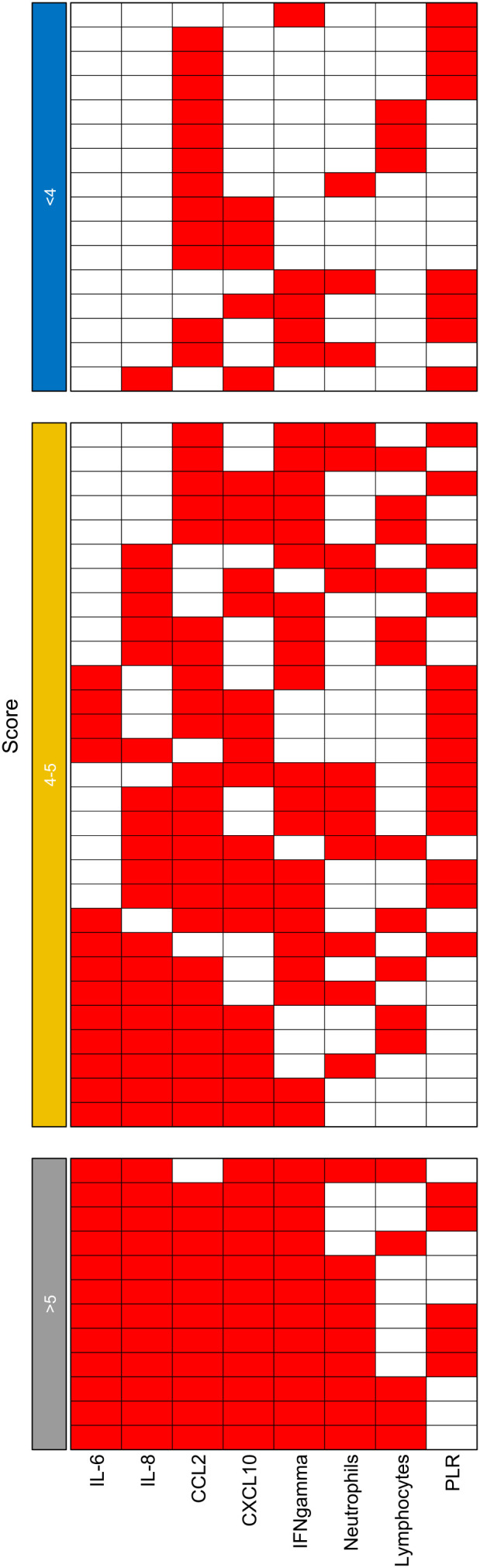
Heatmap of the distribution of the 57 patients in the cohort A stratified by ISBIS grouped calculated according to optimized cut-offs levels. PLR, platelet-to-lymphocyte ratio.

Based on the significant correlations emerging from the first round of analysis we proceeded by building an Immune-suppressive blood index score (ISBIS) score aiming to identify patients with unfavorable outcome of ICI therapy. The ISBIS score was composed by above cutoff baseline levels of neutrophils, CXCL10 IL-6, IL-8 and below cutoff baseline levels of IFN-gamma, lymphocytes, PLR, CCL2 (**Table 3**; **Supplementary Figure 7**), and was able to cluster patients from cohort A into 3 groups with progressively worsening PFS and OS (**Figure 1**).

**Table 3:** Composition of the constructed score and identified variable-specific cutoff values.

Variable	Direction	Threshold
Neutrophils	>	0.047
IFN-Gamma	<	-0.033
CXCL10	>	-0.540
Lymphocytes	<	-0.439
IL-6	>	-0.312
PLR	<	-0.209
CCL2	<	0.449
IL-8	>	-0.320

Each variable contributes to the score based on its directionality and threshold. Variables with a “>“ direction indicate that values above the threshold contribute to the score, while those with a “<“ direction indicate that values below the threshold contribute. PLR, platelet-to-lymphocyte ratio.

**Figure 2:**
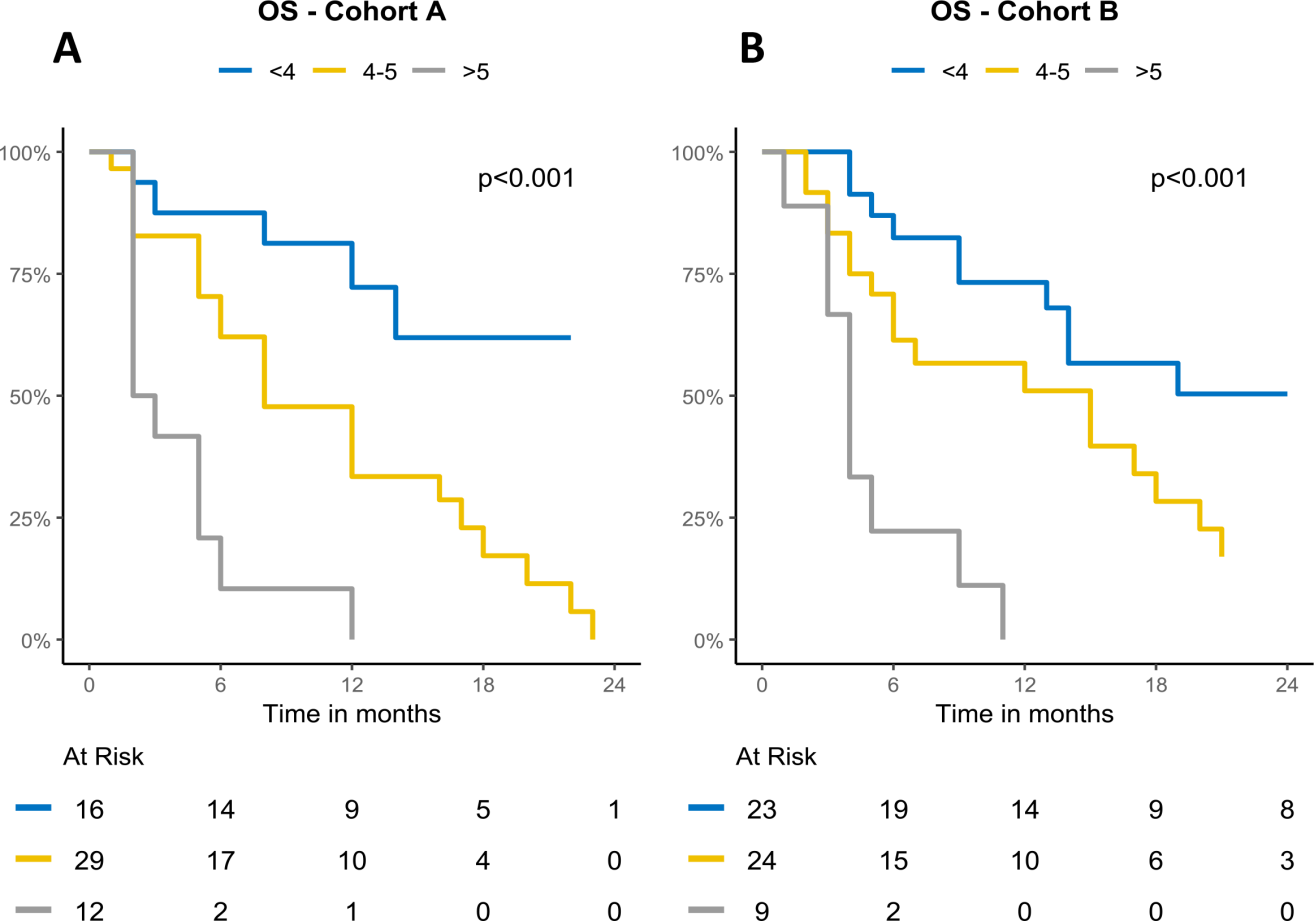
Kaplan–Meier curves of OS in Cohorts A and B, stratified by ISBIS into three categories: <4, 4–5, and >5. **(A)** Overall survival in Cohort A. **(B)** Overall survival in Cohort B.

**Figure 3:**
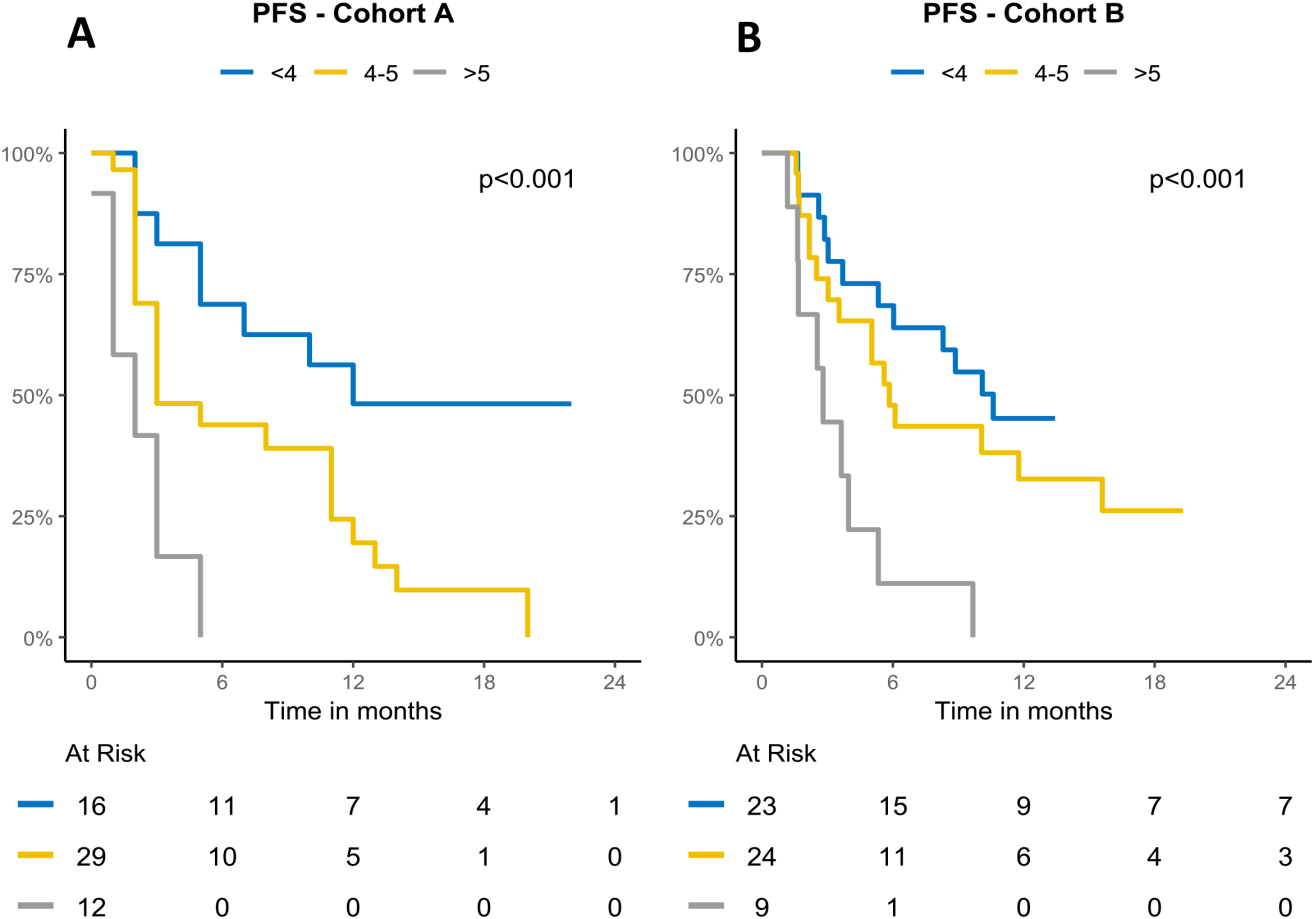
Kaplan–Meier curves of PFS in Cohorts A and B, stratified by ISBIS into three categories: <4, 4–5, and >5. **(A)** Progression free survival in Cohort A. **(B)** Progression free survival in Cohort B.

In detail, patients with an index score <4 (i.e. with none or less than 4 altered parameter; n= 16) displayed a median PFS of 12 months (95% CIs= 5-NA) and median OS not reached (95% CIs= 14-NA); in contrast patients with score 4-5 (four or five altered parameters; n=29) showed a median PFS of 3 months (95% CIs= 3-12) and median OS of 8 months (95% IC = 6-18); finally patients with score >6 (six or more altered parameters; n=12) showed a median PFS of 2 months (95% CIs= 1-NA) and median OS of 2.5 months (95% IC = 2-NA). The differences among both PFS and OS Kaplan Meyers curves across the different subgroups were statistically significant (p < 0.0001). (**Figures 2A**, **3A**; **Tables 4**, **5**).

**Table 4:** Results of the univariate Cox model analysis of ISBIS on OS in Cohorts A and B.

OS	Cohort A		Cohort B	
Score	HR (95%IC)	pvalue	HR (95%IC)	pvalue
4–5 vs <4	3,93 (1,48-10,47)	0,0059	2,88 (1,27-6,52)	0,0109
>5 vs <4	14,02 (4,51-43,58)	<0,0001	8,58 (3,17-23,28)	<0,0001

**Table 5:** Results of the univariate Cox model analysis of ISBIS on PFS in Cohorts A and B.

PFS	Cohort A		Cohort B	
Score	HR (95%IC)	pvalue	HR (95%IC)	pvalue
4–5 vs <4	2,49 (1,14-5,45)	0,0218	1,60 (0,76-3,40)	0,2147
>5 vs <4	9,59 (3,57-25.78)	<0,0001	4,87 (1,97-12,04)	0,0006

The same ISBIS score was then tested and validated in the cohort B population. In detail, patients with score <4 (i.e. with none or less than 4 altered parameter; n= 23) displayed a median PFS of 10.6 months (95% CIs= 6.03-NA) and median OS not reached (95% CIs= 14-NA); in contrast patients with score 4-5 (four or five altered parameters; n=24) showed a median PFS of 5.83 months (95% CIs= 3.53-NA) and median OS of 15 months (95% IC = 6-21); finally patients with score >6 (six or more altered parameters; n=9) showed a median PFS of 2.8 months (95% CIs= 1.67-NA) and median OS of 4 months (95% IC = 3-NA). The differences among both PFS and OS Kaplan Meyers curves across the different subgroups were statistically significant (p = 0.001, p < 0.0001). (**Figures 2B**, **3B**; **Tables 4**, **5**).

To further validate the biological relevance of our model, we evaluated the correlation between the ISBIS categories (low risk <4 vs. high risk ≥4) and individual systemic immune markers (**Supplementary Figures 8A, B**). At baseline, a high ISBIS significantly correlated with a pro-inflammatory and immunosuppressed environment, characterized by elevated IL-6, IL-8, neutrophils, and PLR, alongside reduced IFN-gamma and lymphocyte counts across both cohorts.

Moreover, longitudinal analysis revealed distinct on-treatment immune modulations based on the ISBIS risk group. While low-risk patients (<4) exhibited a significant decrease in soluble immune checkpoints such as LAG3 and CD80 during therapy, high-risk patients (≥4) were uniquely characterized by a marked on-treatment increase in CXCL10.

### Nomogram integrating ISBIS and key clinical variables improves risk stratification

As final step, a nomogram was constructed using a stepwise regression approach, incorporating the previously developed ISBIS score along with selected clinical variables with acknowledged prognostic implications. The final model retained the NLR, ECOG performance status >2 (PS), disease burden (stage IVB versus IVA), gender, and a composite risk score as significant predictors of clinical outcomes.

**Figure 4:**
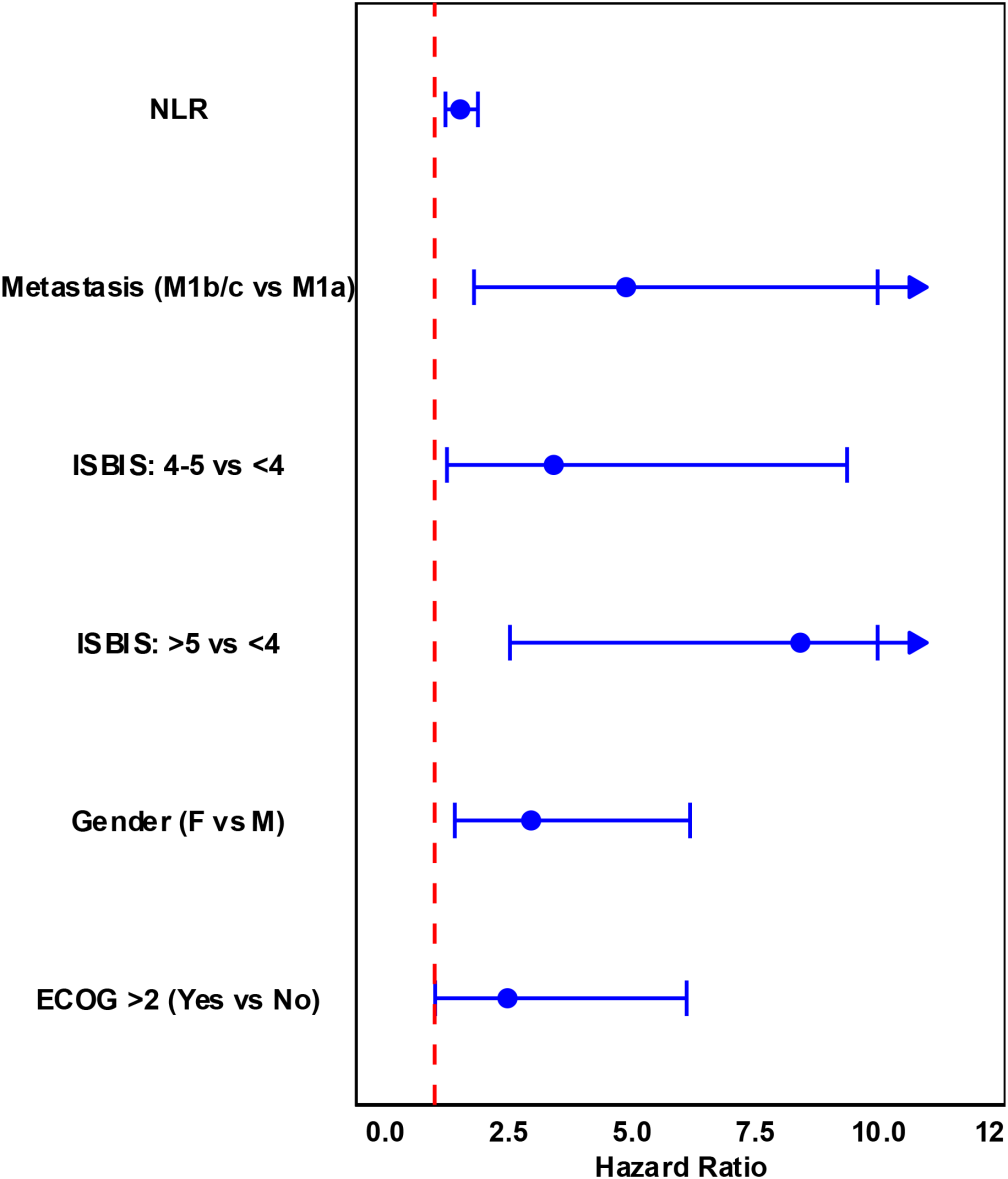
Forest plot of the multivariate Cox proportional hazards model for OS in Cohorts A, showing hazard ratios and 95% confidence intervals for ISBIS and other covariates.

In cohort A, multivariate Cox regression analysis demonstrated that higher NLR value was associated with an increased risk of adverse outcomes (HR: 1.52, 95% CI: 1.23–1.89, p = 0.0001). Similarly, ECOG PS >2 (HR: 2.49, 95% CI: 1.01–6.12, p = 0.0477), stage IVB (HR: 4.90, 95% CI: 1.80–13.32, p = 0.0019), and female gender (HR: 2.96, 95% CI: 1.42–6.19, p = 0.0039) were all significantly associated with poorer prognosis. The composite risk score further stratified patients, with those scoring >5 having a markedly higher hazard ratio (HR: 8.43, 95% CI: 2.54–28.01, p = 0.0005), compared to those with a score <4 (**Table 6**; **Figure 4**).

**Table 6:** Multivariate Cox regression model used to construct the nomogram.

OS	HR (95%IC)	pvalue
NLR	1,52 (1,22-1,88)	0,0001
ECOG >2 (Yes vs No)	2,48 (1,01-6,12)	0,0477
Metastasis (M1b_c vsM1a)	4,89 (1,80-13,32)	0,0019
ISBIS	-	0,0023
4–5 vs <4	3,42 (1,25-9,38)	0,0167
>5 vs <4	8,43 (2,53-28,01)	0,0005
Gender (F vs M)	2,96 (1,41-6,19)	0,0039

Higher neutrophil-to-lymphocyte ratio (NLR), ECOG performance status >2, M1b/c metastatic stage, female gender, and an increasing composite risk score were all significantly associated with worse prognosis in cohort A. OS, overall survival; NLR, neutrophil-to-lymphocyte-ratio; ECOG, Eastern Cooperative Oncology Group; ISBIS, Immune-Suppressive Blood Index Score.

**Figure 5:**
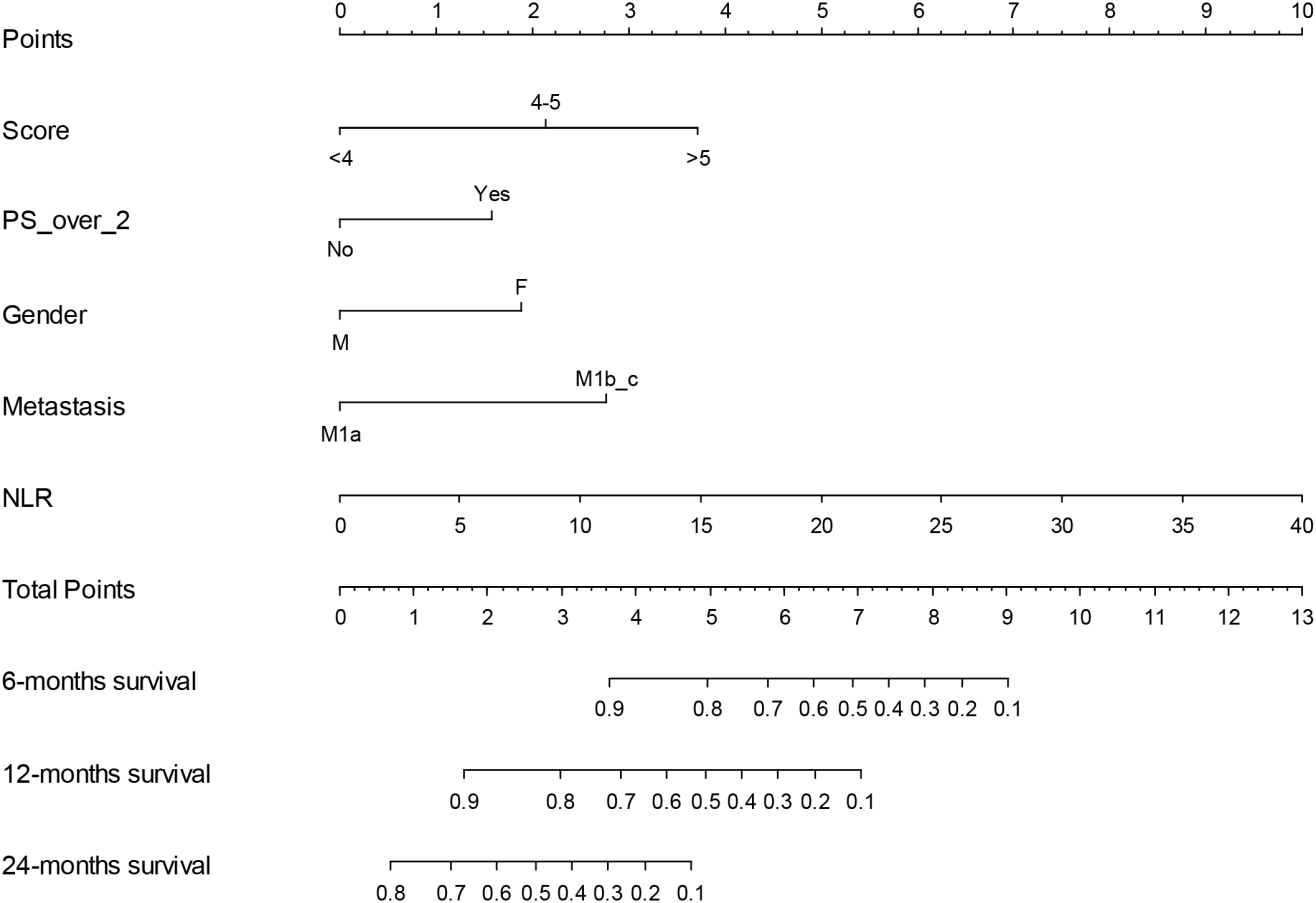
Nomogram for predicting survival in cohort A. The model integrates key prognostic variables, including NLR, ECOG performance status >2, gender, metastatic stage, and composite risk score. Each variable corresponds to a score on the top scale, with total points summing to estimate 6-, 12-, and 24-month survival probabilities.

The nomogram allows individualized survival prediction by assigning a point value to each predictor variable. For a given patient, the value of each variable (e.g., NLR, PS >2, metastatic stage, gender, and ISBIS score) corresponds to a specific number of points on the top “Points” scale. The total points from all variables are then summed and located on the “Total Points” scale. By projecting downward, the total points align with estimated probabilities of 6-month, 12-month, and 24-month survival. Higher total point scores correspond to lower survival probabilities, reflecting higher predicted risk (**Figure 5**).

The discriminative performance of the nomogram in cohort A was assessed using the concordance index (c-index), which demonstrated a high discriminative ability of 0.83, indicating strong reliability in risk stratification.

Validation of the nomogram on cohort B yielded an accuracy of 73.8% (95% CI: 57.9–86.1%), with a sensitivity of 78.1% and specificity of 60.0%. The positive predictive value (PPV) was 86.2%, while the negative predictive value (NPV) was 46.2%. The balanced accuracy was 69.1%, and the kappa statistic indicated moderate agreement (κ = 0.35).

## Discussion

In this study, we identified a baseline systemic Immune-Suppressive Blood Index Score (ISBIS), composed of above-cutoff baseline levels of neutrophils, CXCL10, IL-6, and IL-8, and below-cutoff baseline levels of IFN-γ, lymphocytes, PLR and CCL2, which correlates with poor outcomes in advanced NSCLC patients receiving anti-PD-1 therapy. These findings align with mounting evidence that a pro-inflammatory, myeloid-dominated immune milieu undermines ICI efficacy, whereas robust T cell-driven immunity supports improved prognosis. Importantly, each component of the ISBIS score is supported by previously published literature, reinforcing the biological plausibility of the composite model. Elevated neutrophil counts, included in the ISBIS high-risk profile, have consistently been associated with poor outcomes in NSCLC patients treated with ICIs. Increased neutrophils reflect a myeloid-dominated immune landscape characterized by expansion of myeloid-derived suppressor cells (MDSCs), secretion of immunosuppressive cytokines and inhibition of cytotoxic T cell activity (10, 11). Similarly, reduced lymphocyte counts, also included in the ISBIS high-risk configuration, reflect impaired adaptive immune competence and reduced T cell-mediated antitumor responses. Several studies have demonstrated that lymphopenia is associated with inferior survival in patients treated with ICIs, highlighting the importance of preserved lymphocyte populations for effective immune checkpoint blockade (12, 13). The inclusion of elevated IL-6 and IL-8 in the high-risk ISBIS profile further supports the role of systemic inflammation in immunotherapy resistance. Our finding of high baseline IL-6 as a negative prognostic factor is supported by a recent meta-analysis showing that elevated IL-6 was significantly associated with worse progression-free survival in NSCLC patients treated with PD-1 inhibitors (14). Additionally, in chemo-immunotherapy-treated NSCLC, baseline and dynamic increases in IL-6 have been associated with poor survival (15, 16), while on-treatment decrease of plasma IL-6 predicted improved clinical outcomes and the extent of change in IL-6 differed between best overall response categories (17). Similarly, altered IL-8 has been identified as part of an immunosuppressive circulating profile, with elevated plasma IL-8 levels associated with poor response to anti-PD-1 therapy in NSCLC patients (18, 19). Both cytokines promote tumor progression through recruitment of immunosuppressive myeloid populations, induction of angiogenesis and inhibition of cytotoxic immune responses.

CXCL10, included as elevated in the high-risk group, reflects a more complex immunological role. The dynamic increase in CXCL10 (IP-10) during ICI therapy among patients with early progression in our cohorts is particularly notable. CXCL10 is often induced by IFN signaling and can recruit T cells. However, chronic or dysregulated expression may also reflect dysfunctional immune activation. In line with this biological rationale, a large-scale study in ICI-treated NSCLC patients found that both pre-treatment and on-treatment changes in chemokines, including CXCL10, were associated with survival outcomes (20). These findings support the hypothesis that both the presence and kinetics of CXCL10 may meaningfully reflect immune dynamics during ICI therapy (21). Conversely, reduced IFN-γ levels, also part of the high-risk ISBIS configuration, are consistent with impaired antitumor immune activation. IFN-γ plays a central role in antigen presentation, cytotoxic T cell activation and immune surveillance, and higher circulating IFN-γ levels have been associated with improved response to PD-1 blockade (22). Reduced IFNγ levels therefore likely reflect impaired immune activation and diminished responsiveness to immunotherapy.

Interestingly, reduced PLR and reduced CCL2 were also retained in the ISBIS model, highlighting the synergistic nature of the AIM-based algorithm. Within this integrated multivariable framework, each parameter contributes to the overall risk profile based on its combined interaction with other immune variables rather than isolated univariate associations. This underscores the importance of composite immune signatures, which may capture complex systemic immune dynamics more accurately than single biomarkers alone.

The paradoxical role of cytokines and chemokines in the tumor microenvironment is increasingly recognized. These mediators can both support antitumor immunity and promote tumor progression, depending on context, timing and cellular sources (23, 24). This duality complicates interpretation of systemic chemokine changes, particularly in the setting of ICI therapy, and further supports the value of composite indices such as ISBIS. As a less explored finding, we observed that soluble PD-L2 levels decrease on treatment in patients with poor outcomes. While data on soluble PD-L2 in NSCLC remain limited, this observation may reflect therapy-induced changes in antigen-presenting cells, checkpoint ligand redistribution, or immune cell remodeling. Pre-analytic studies also indicate that assay type and sample matrix significantly affect detection of soluble checkpoint molecules (25–28), highlighting the need for standardized methodologies.

Our Immune-Suppressive Blood Index Score integrates baseline immune characteristics and offers a more holistic approach than single biomarkers for assessing systemic immune status. Correlation analyses confirmed that ISBIS stratification captures distinct baseline inflammatory profiles and identifies specific on-treatment immune modulations, such as CXCL10 increases in high-risk patients. When combined with clinical predictors including ECOG status, metastatic stage, sex and NLR, ISBIS further improved risk stratification and individualized prognostic estimation. These findings are consistent with recent work demonstrating that circulating immune mediators measured before and during therapy are associated with survival in ICI-treated NSCLC. For example, a cohort of 183 NSCLC patients profiled for 73 soluble mediators identified multiple immune factors, including CXCL10, independently associated with survival outcomes (20). Similarly, recent neoadjuvant studies have shown that early transcriptomic and cellular changes in peripheral blood during PD-1 blockade correlate with response (29, 30), supporting the concept that systemic immune adaptation reflects therapeutic outcome. Given the growing recognition of cytokines and chemokines as both biomarkers and therapeutic targets, there is increasing interest in combining ICI therapy with cytokine-modulating agents. IL-6 and IL-8 blockade are currently being explored in preclinical and early clinical settings as adjuncts to immunotherapy (31–34). Our findings support this strategy, particularly for patients with high ISBIS scores, who may benefit from targeted interventions aimed at reducing systemic inflammation and restoring immune balance.

One of the key strengths of our study lies in the use of two independent real-world cohorts, including a discovery and an external validation cohort, enhancing robustness and reproducibility. Despite differences in clinical characteristics, ISBIS maintained its discriminatory ability across both populations, supporting its generalizability. Another strength is the integrative multidimensional approach combining circulating cytokines and routine hematologic parameters, which captures the complexity of systemic immune regulation. The use of minimally invasive peripheral blood biomarkers further enhances clinical applicability. Finally, integration of ISBIS into a nomogram with established clinical variables increases translational relevance and supports personalized treatment decision-making.

Our study has several limitations. First, its retrospective nature and relatively small sample sizes limit generalizability. Second, longitudinal sampling was limited to specific time points. Third, prospective validation in larger cohorts is required. Fourth, toxicity data were not available. Finally, mechanistic studies are needed to clarify the biological sources of circulating mediators. Future work should focus on prospective validation of ISBIS in multi-center cohorts and integration with tissue-based biomarkers, imaging, and advanced modeling approaches. Emerging multimodal models combining imaging, blood biomarkers, and clinical data have already demonstrated high predictive accuracy in ICI-treated populations (35, 36). Mechanistic studies using single-cell approaches may further elucidate the biological origin of ISBIS components and guide targeted therapeutic interventions.

In conclusion, our study highlights the critical impact of systemic inflammation and immune suppression on ICI outcomes in NSCLC. The ISBIS score captures this complex interplay in a clinically meaningful way, stratifying patients into risk groups and guiding prognostic estimation. Incorporation of blood-based immunologic profiling into clinical practice may enhance personalized immunotherapy strategies and support adaptive treatment approaches in advanced NSCLC.

## Data Availability

All relevant data is contained within the article: The original contributions presented in the study are included in the article/**Supplementary Material**, further inquiries can be directed to the corresponding author/s.
