# Supplementary material for: A blood-based immune-suppressive index stratifies immunotherapy outcomes in advanced NSCLC
Source: Front Immunol. 2026 May 8;17:1772319. doi: 10.3389/fimmu.2026.1772319 (PMC13194465; doi:10.3389/fimmu.2026.1772319)
Supplement: Supplementary file 1 [file Presentation1.pptx]

## Slide 1
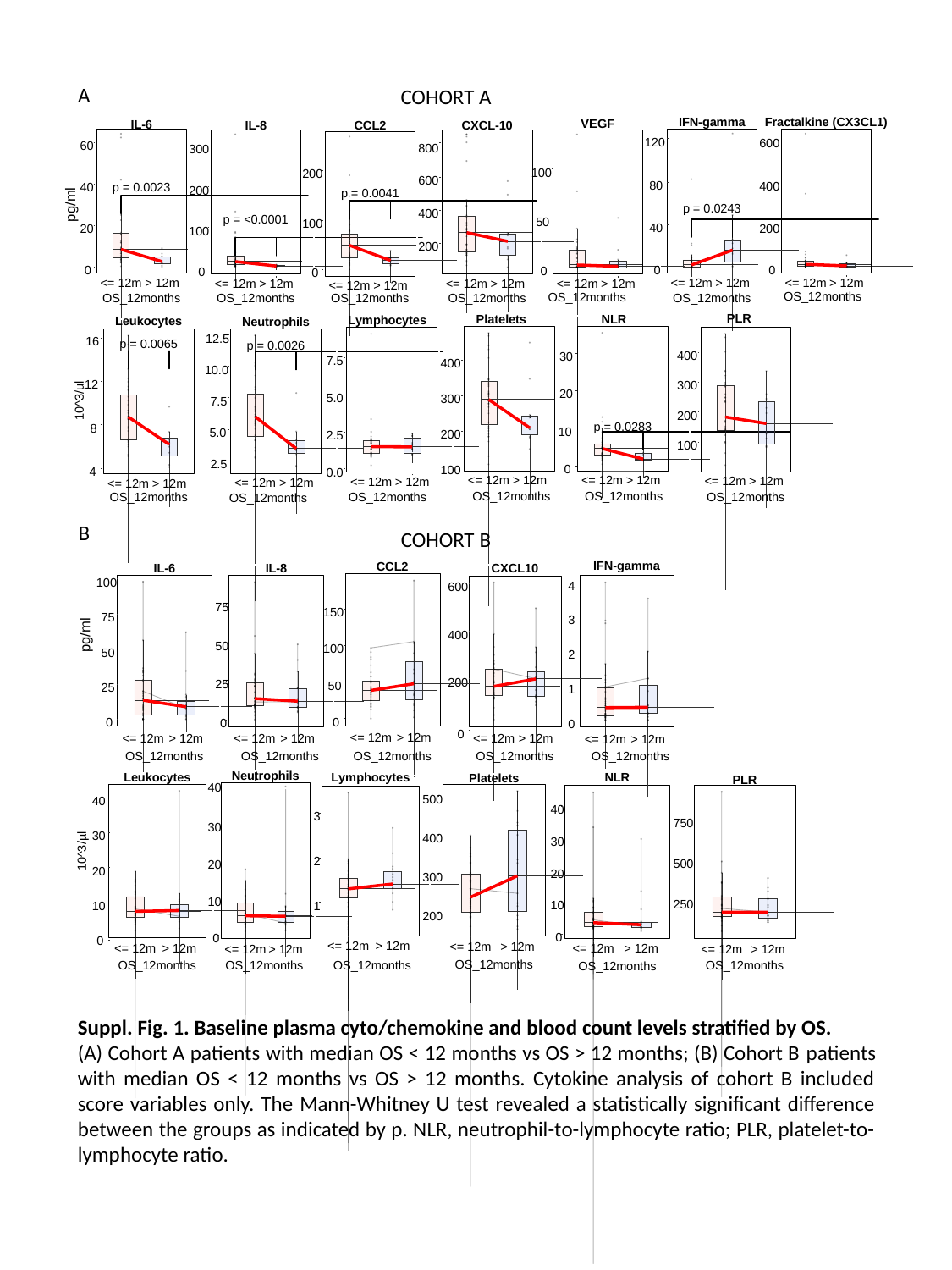

A
COHORT A
IFN-gamma
Fractalkine (CX3CL1)
IL-6
VEGF
IL-8
CXCL-10
CCL2
120
600
60
800
300
100
200
600
80
400
40
p = 0.0023
200
pg/ml
p = 0.0041
p = 0.0243
400
50
p = <0.0001
100
40
200
20
100
200
0
0
0
0
0
0
<= 12m
> 12m
<= 12m
> 12m
<= 12m
> 12m
<= 12m
> 12m
<= 12m
> 12m
<= 12m
> 12m
<= 12m
> 12m
OS_12months
OS_12months
OS_12months
OS_12months
OS_12months
OS_12months
OS_12months
Platelets
PLR
NLR
Lymphocytes
Leukocytes
Neutrophils
12.5
16
p = 0.0065
p = 0.0026
400
30
7.5
400
10.0
12
300
10^3/µl
20
5.0
300
7.5
200
8
p = 0.0283
10
5.0
200
2.5
100
2.5
0
100
4
0.0
<= 12m
> 12m
<= 12m
> 12m
<= 12m
> 12m
<= 12m
> 12m
<= 12m
> 12m
<= 12m
> 12m
OS_12months
OS_12months
OS_12months
OS_12months
OS_12months
OS_12months
B
COHORT B
IFN-gamma
CCL2
CXCL10
IL-6
IL-8
100
4
600
75
150
75
3
pg/ml
400
50
100
50
2
200
25
50
25
1
0
0
0
0
0
<= 12m
> 12m
<= 12m
> 12m
<= 12m
> 12m
<= 12m
> 12m
<= 12m
> 12m
OS_12months
OS_12months
OS_12months
OS_12months
OS_12months
Neutrophils
Leukocytes
Lymphocytes
NLR
40
30
20
10
0
<= 12m
> 12m
OS_12months
Platelets
500
400
300
200
<= 12m
> 12m
OS_12months
PLR
40
40
3
750
30
30
10^3/µl
2
500
20
20
10
250
1
10
0
0
<= 12m
> 12m
<= 12m
> 12m
<= 12m
> 12m
<= 12m
> 12m
OS_12months
OS_12months
OS_12months
OS_12months
Suppl. Fig. 1. Baseline plasma cyto/chemokine and blood count levels stratified by OS.
(A) Cohort A patients with median OS < 12 months vs OS > 12 months; (B) Cohort B patients with median OS < 12 months vs OS > 12 months. Cytokine analysis of cohort B included score variables only. The Mann-Whitney U test revealed a statistically significant difference between the groups as indicated by p. NLR, neutrophil-to-lymphocyte ratio; PLR, platelet-to-lymphocyte ratio.

## Slide 2
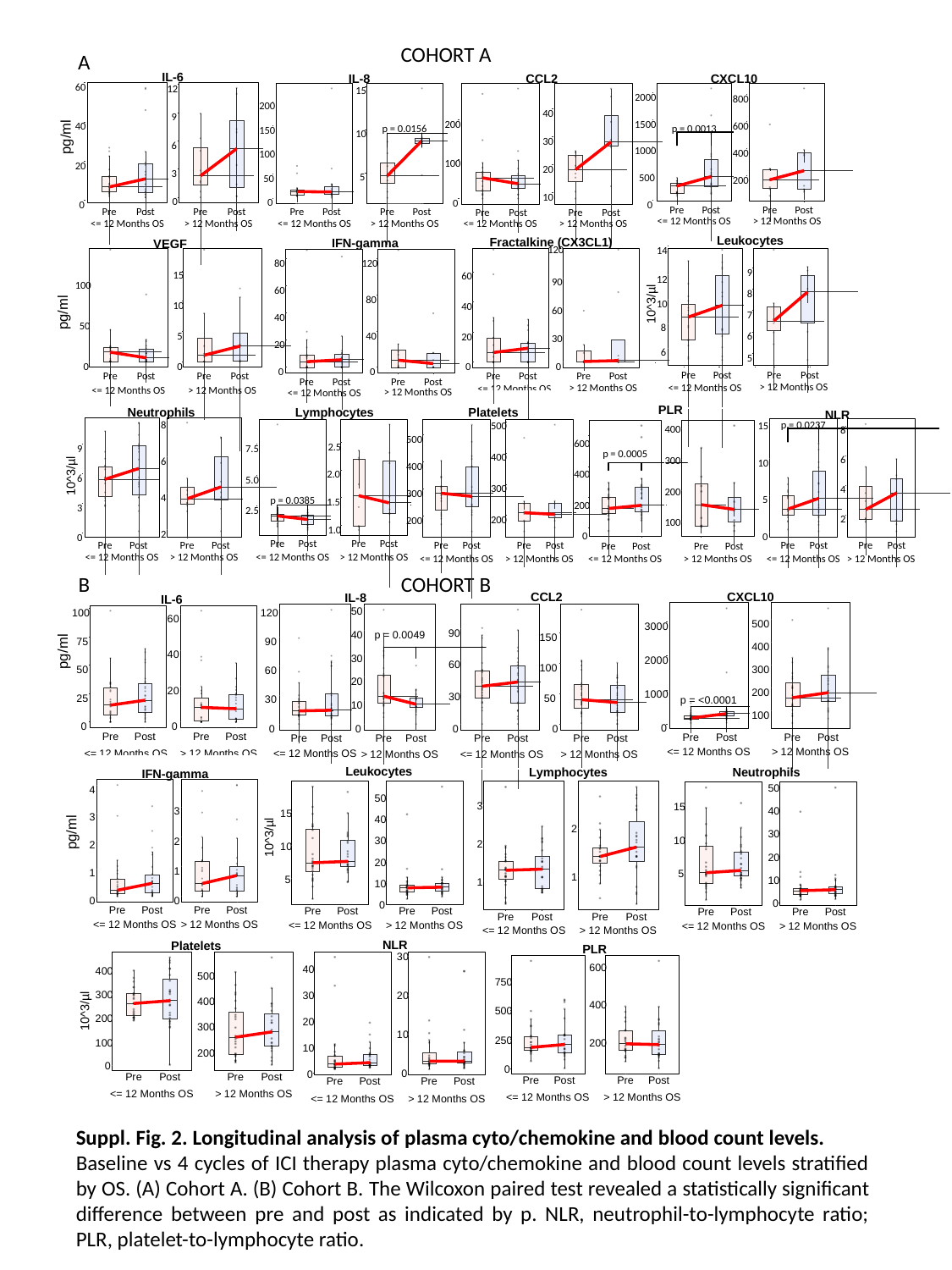

COHORT A
A
IL-6
CXCL10
CCL2
IL-8
60
12
15
2000
800
200
40
9
1500
200
600
40
pg/ml
150
p = 0.0156
p = 0.0013
10
30
6
1000
400
100
100
20
20
3
5
500
50
200
10
0
0
0
0
0
Pre
Post
Pre
Post
Pre
Post
Pre
Post
Pre
Post
Pre
Post
Pre
Post
Pre
Post
<= 12 Months OS
> 12 Months OS
<= 12 Months OS
> 12 Months OS
<= 12 Months OS
> 12 Months OS
<= 12 Months OS
> 12 Months OS
Leukocytes
IFN-gamma
Fractalkine (CX3CL1)
VEGF
120
14
9
12
8
10
7
8
6
6
5
80
120
15
60
90
100
60
10^3/µl
80
pg/ml
10
40
60
40
50
40
5
20
30
20
0
0
0
0
0
0
Post
Post
Pre
Pre
> 12 Months OS
<= 12 Months OS
Pre
Post
Pre
Post
Pre
Post
Pre
Pre
Post
Pre
Post
Pre
Post
Pre
Post
> 12 Months OS
<= 12 Months OS
<= 12 Months OS
> 12 Months OS
> 12 Months OS
<= 12 Months OS
Lymphocytes
NLR
15
p = 0.0237
8
6
10
4
5
2
0
Pre
Post
Pre
Post
<= 12 Months OS
> 12 Months OS
Neutrophils
Platelets
PLR
400
600
p = 0.0005
300
400
200
200
100
0
Pre
Post
Pre
Post
<= 12 Months OS
> 12 Months OS
8
500
500
2.5
9
7.5
400
6
400
10^3/µl
2.0
6
5.0
300
300
4
1.5
p = 0.0385
3
2.5
200
200
1.0
2
0
Pre
Post
Pre
Post
Pre
Post
Pre
Post
Pre
Post
Pre
Post
<= 12 Months OS
> 12 Months OS
<= 12 Months OS
> 12 Months OS
<= 12 Months OS
> 12 Months OS
COHORT B
B
IL-6
100
60
75
40
50
20
25
0
0
Pre
Post
Pre
Post
<= 12 Months OS
> 12 Months OS
CXCL10
CCL2
IL-8
50
120
40
p = 0.0049
90
30
60
20
30
10
0
0
Pre
Post
Pre
Post
<= 12 Months OS
> 12 Months OS
500
3000
90
150
pg/ml
400
2000
60
100
300
200
1000
30
50
p = <0.0001
100
0
0
0
Pre
Post
Pre
Post
Pre
Post
Pre
Post
<= 12 Months OS
<= 12 Months OS
> 12 Months OS
> 12 Months OS
IFN-gamma
4
3
3
2
2
1
1
0
0
Pre
Post
Pre
Post
<= 12 Months OS
> 12 Months OS
Leukocytes
50
15
40
30
10
20
5
10
0
Pre
Post
Pre
Post
<= 12 Months OS
> 12 Months OS
Neutrophils
50
15
40
30
10
20
5
10
0
Pre
Post
Pre
Post
<= 12 Months OS
> 12 Months OS
Lymphocytes
3
2
2
1
1
Pre
Post
Pre
Post
<= 12 Months OS
> 12 Months OS
pg/ml
10^3/µl
NLR
30
40
30
20
20
10
10
0
0
Pre
Post
Pre
Post
<= 12 Months OS
> 12 Months OS
Platelets
400
500
300
400
200
300
100
200
0
Pre
Post
Pre
Post
<= 12 Months OS
> 12 Months OS
PLR
600
750
400
500
250
200
0
Pre
Post
Pre
Post
<= 12 Months OS
> 12 Months OS
10^3/µl
Suppl. Fig. 2. Longitudinal analysis of plasma cyto/chemokine and blood count levels.
Baseline vs 4 cycles of ICI therapy plasma cyto/chemokine and blood count levels stratified by OS. (A) Cohort A. (B) Cohort B. The Wilcoxon paired test revealed a statistically significant difference between pre and post as indicated by p. NLR, neutrophil-to-lymphocyte ratio; PLR, platelet-to-lymphocyte ratio.

## Slide 3
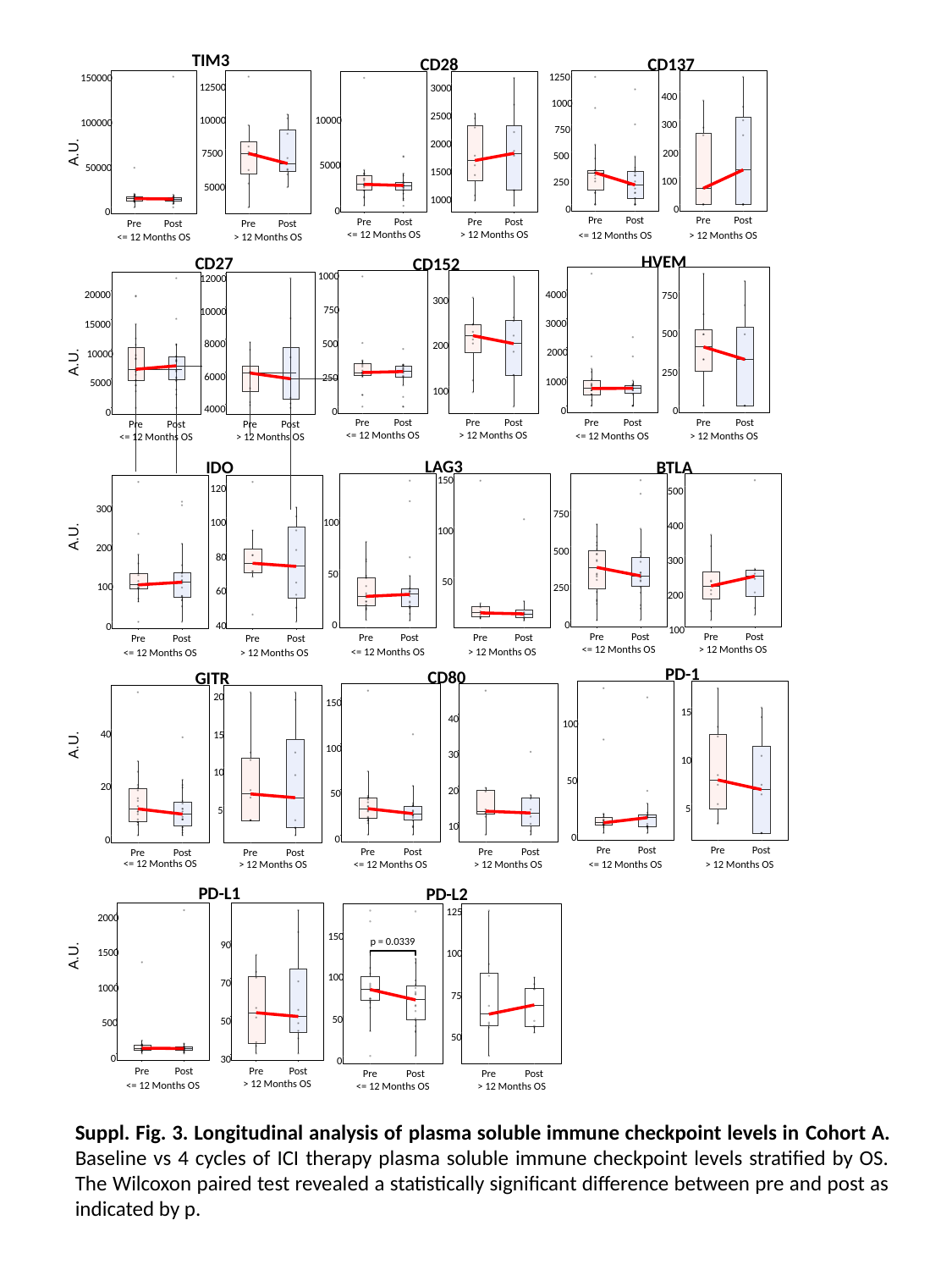

TIM3
150000
12500
10000
100000
7500
50000
5000
0
Pre
Post
Pre
Post
<= 12 Months OS
> 12 Months OS
CD28
3000
2500
10000
2000
5000
1500
1000
0
Pre
Post
Pre
Post
<= 12 Months OS
> 12 Months OS
CD137
1250
400
1000
300
750
200
500
100
250
0
0
Pre
Post
Pre
Post
<= 12 Months OS
> 12 Months OS
A.U.
HVEM
4000
750
3000
500
2000
250
1000
0
0
Pre
Post
Pre
Post
<= 12 Months OS
> 12 Months OS
CD152
1000
300
750
500
200
250
100
0
Pre
Post
Pre
Post
<= 12 Months OS
> 12 Months OS
CD27
12000
20000
10000
15000
8000
10000
6000
5000
4000
0
Pre
Post
Pre
Post
<= 12 Months OS
> 12 Months OS
A.U.
BTLA
500
750
400
500
300
250
200
0
100
Pre
Post
Pre
Post
<= 12 Months OS
> 12 Months OS
LAG3
150
100
100
50
50
0
Pre
Post
Pre
Post
<= 12 Months OS
> 12 Months OS
IDO
120
300
100
200
80
100
60
40
0
Pre
Post
Pre
Post
<= 12 Months OS
> 12 Months OS
A.U.
PD-1
15
100
10
50
5
0
Pre
Post
Pre
Post
<= 12 Months OS
> 12 Months OS
CD80
150
40
100
30
20
50
10
0
Pre
Post
Pre
Post
<= 12 Months OS
> 12 Months OS
GITR
20
40
15
10
20
5
0
Pre
Post
Pre
Post
<= 12 Months OS
> 12 Months OS
A.U.
PD-L1
2000
90
1500
70
1000
50
500
0
30
Pre
Post
Pre
Post
> 12 Months OS
<= 12 Months OS
PD-L2
125
150
p = 0.0339
100
100
75
50
50
0
Pre
Post
Pre
Post
<= 12 Months OS
> 12 Months OS
A.U.
Suppl. Fig. 3. Longitudinal analysis of plasma soluble immune checkpoint levels in Cohort A. Baseline vs 4 cycles of ICI therapy plasma soluble immune checkpoint levels stratified by OS. The Wilcoxon paired test revealed a statistically significant difference between pre and post as indicated by p.

## Slide 4
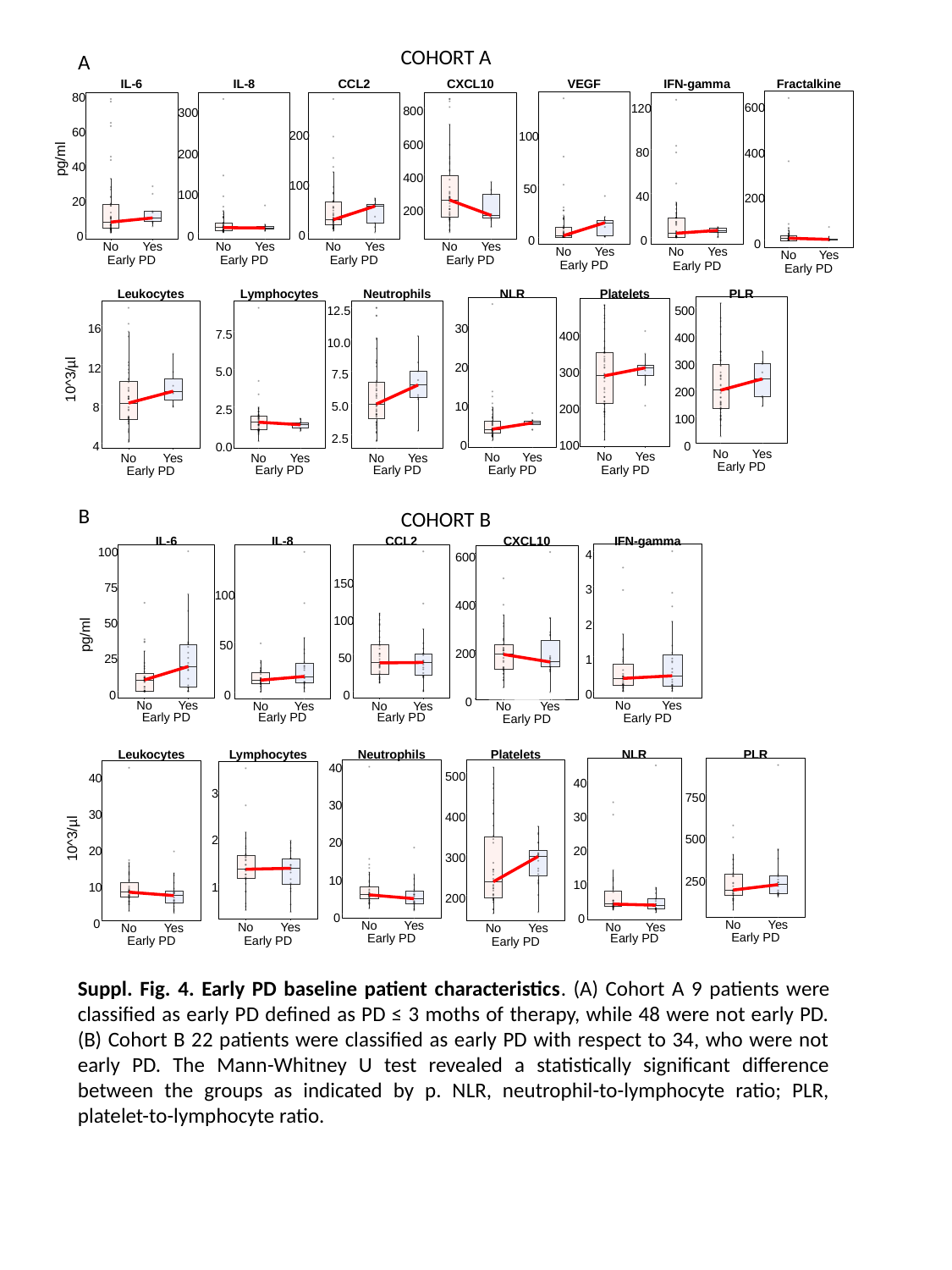

COHORT A
A
IL-6
80
60
40
20
0
No
Yes
Early PD
IL-8
300
200
100
0
No
Yes
Early PD
CCL2
200
100
0
No
Yes
Early PD
CXCL10
800
600
400
200
No
Yes
Early PD
VEGF
100
50
0
No
Yes
Early PD
IFN-gamma
120
80
40
0
No
Yes
Early PD
Fractalkine
600
400
200
0
No
Yes
Early PD
pg/ml
Leukocytes
16
12
8
4
No
Yes
Early PD
Lymphocytes
7.5
5.0
2.5
0.0
No
Yes
Early PD
Neutrophils
12.5
10.0
7.5
5.0
2.5
No
Yes
Early PD
NLR
30
20
10
0
Yes
No
Early PD
Platelets
400
300
200
100
No
Yes
Early PD
PLR
500
400
300
200
100
0
No
Yes
Early PD
10^3/µl
B
COHORT B
IL-6
100
75
50
25
0
No
Yes
Early PD
IL-8
100
50
0
No
Yes
Early PD
CCL2
150
100
50
0
No
Yes
Early PD
CXCL10
600
400
200
0
No
Yes
Early PD
IFN-gamma
4
3
2
1
0
No
Yes
Early PD
pg/ml
Leukocytes
40
30
20
10
0
No
Yes
Early PD
Lymphocytes
3
2
1
No
Yes
Early PD
Neutrophils
40
30
20
10
0
No
Yes
Early PD
Platelets
500
400
300
200
No
Yes
Early PD
NLR
40
30
20
10
0
No
Yes
Early PD
PLR
750
500
250
No
Yes
Early PD
10^3/µl
Suppl. Fig. 4. Early PD baseline patient characteristics. (A) Cohort A 9 patients were classified as early PD defined as PD ≤ 3 moths of therapy, while 48 were not early PD. (B) Cohort B 22 patients were classified as early PD with respect to 34, who were not early PD. The Mann-Whitney U test revealed a statistically significant difference between the groups as indicated by p. NLR, neutrophil-to-lymphocyte ratio; PLR, platelet-to-lymphocyte ratio.

## Slide 5
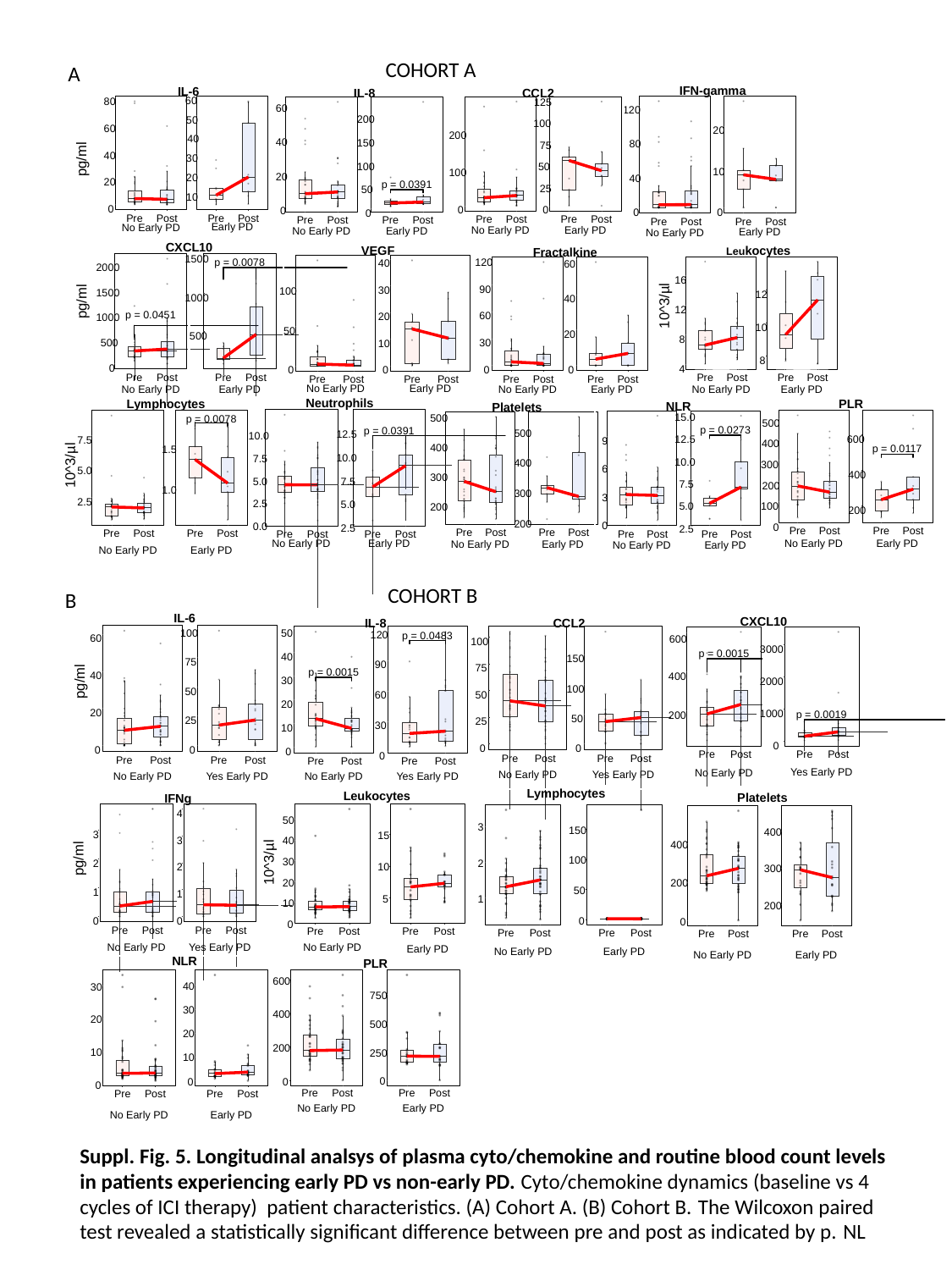

COHORT A
A
IL-6
60
80
50
60
40
40
30
20
20
10
0
Pre
Post
Pre
Post
Early PD
No Early PD
IFN-gamma
120
20
80
10
40
0
0
Pre
Post
Pre
Post
Early PD
No Early PD
IL-8
60
200
40
150
100
20
p = 0.0391
50
0
0
Pre
Post
Pre
Post
No Early PD
Early PD
CCL2
125
100
200
75
50
100
25
0
0
Pre
Post
Pre
Post
No Early PD
Early PD
pg/ml
CXCL10
1500
p = 0.0078
2000
1500
1000
1000
p = 0.0451
500
500
0
Pre
Post
Pre
Post
No Early PD
Early PD
VEGF
40
30
100
20
50
10
0
0
Pre
Post
Pre
Post
No Early PD
Early PD
Leukocytes
16
12
12
10
8
8
4
Pre
Post
Pre
Post
No Early PD
Early PD
Fractalkine
120
60
90
40
60
20
30
0
0
Pre
Post
Pre
Post
No Early PD
Early PD
pg/ml
10^3/µl
Neutrophils
p = 0.0391
12.5
10.0
7.5
5.0
2.5
Post
Pre
Post
Early PD
10.0
7.5
5.0
2.5
0.0
Pre
No Early PD
Lymphocytes
p = 0.0078
7.5
1.5
5.0
1.0
2.5
Post
Pre
Post
Pre
No Early PD
Early PD
PLR
500
600
400
p = 0.0117
300
400
200
100
200
0
Pre
Post
Pre
Post
No Early PD
Early PD
NLR
15.0
p = 0.0273
12.5
9
10.0
6
7.5
3
5.0
0
2.5
Pre
Post
Pre
Post
No Early PD
Early PD
Platelets
500
500
400
400
300
300
200
200
Pre
Post
Pre
Post
No Early PD
Early PD
10^3/µl
COHORT B
B
CXCL10
600
3000
p = 0.0015
400
2000
1000
200
p = 0.0019
0
Pre
Post
Pre
Post
Yes Early PD
No Early PD
IL-6
100
60
75
40
50
20
25
0
0
Pre
Post
Pre
Post
No Early PD
Yes Early PD
IL-8
50
120
p = 0.0483
40
90
p = 0.0015
30
60
20
30
10
0
0
Pre
Post
Pre
Post
Yes Early PD
No Early PD
CCL2
100
150
75
100
50
50
25
0
0
Pre
Post
Pre
Post
Yes Early PD
No Early PD
pg/ml
IFNg
4
3
3
2
2
1
1
0
0
Pre
Post
Pre
Post
No Early PD
Yes Early PD
Lymphocytes
3
150
100
2
50
1
0
Pre
Post
Pre
Post
No Early PD
Early PD
Leukocytes
50
15
40
30
10
20
5
10
0
Pre
Post
Pre
Post
No Early PD
Early PD
Platelets
400
400
300
200
200
0
Pre
Post
Pre
Post
No Early PD
Early PD
pg/ml
10^3/µl
NLR
40
30
30
20
20
10
10
0
0
Pre
Post
Pre
Post
No Early PD
Early PD
PLR
600
750
400
500
200
250
0
0
Pre
Post
Pre
Post
No Early PD
Early PD
Suppl. Fig. 5. Longitudinal analsys of plasma cyto/chemokine and routine blood count levels in patients experiencing early PD vs non-early PD. Cyto/chemokine dynamics (baseline vs 4 cycles of ICI therapy) patient characteristics. (A) Cohort A. (B) Cohort B. The Wilcoxon paired test revealed a statistically significant difference between pre and post as indicated by p. NL

## Slide 6
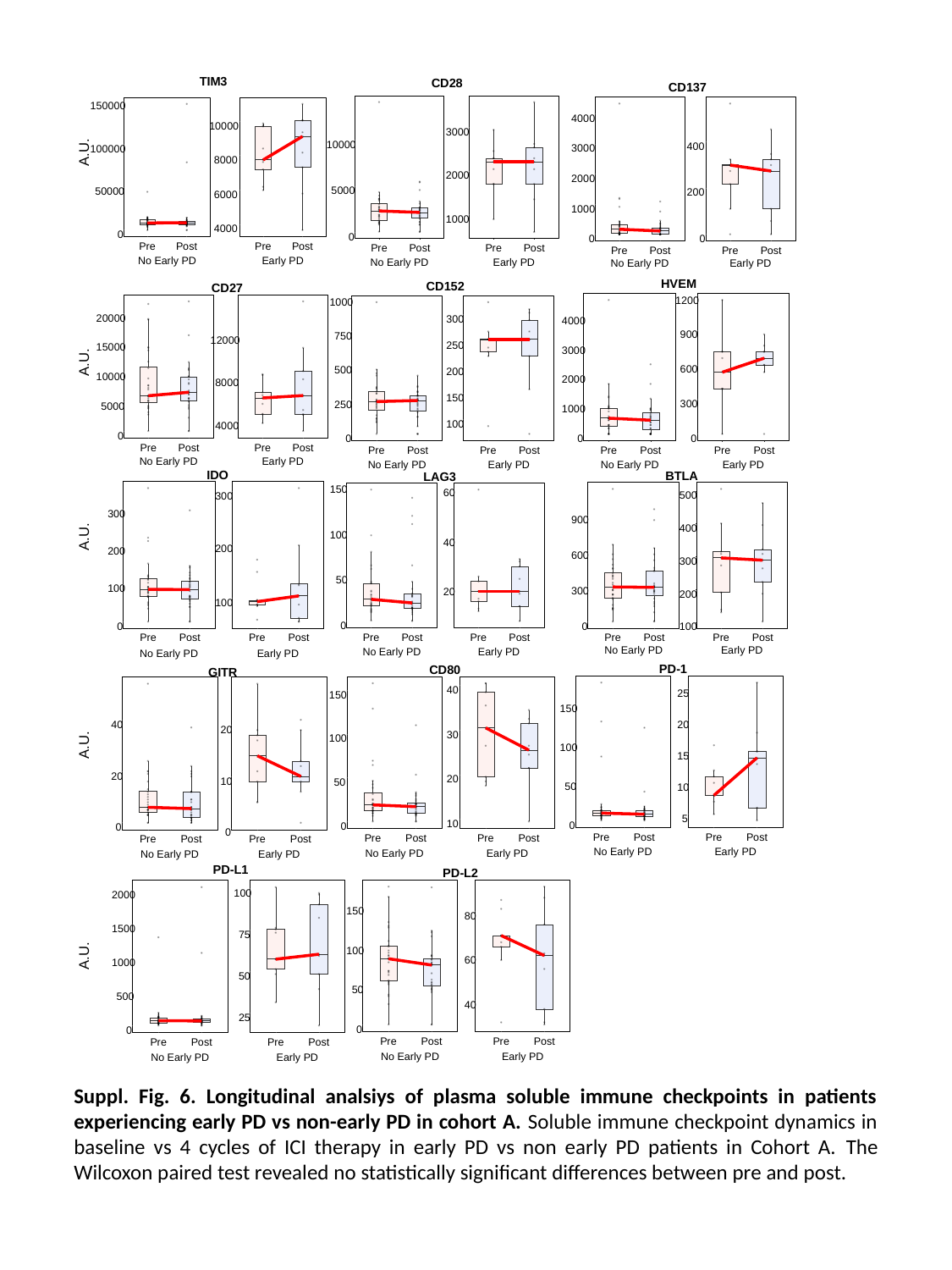

TIM3
150000
10000
100000
8000
50000
6000
4000
0
Pre
Post
Pre
Post
No Early PD
Early PD
CD28
3000
10000
2000
5000
1000
0
Pre
Post
Pre
Post
No Early PD
Early PD
CD137
4000
400
3000
2000
200
1000
0
0
Pre
Post
Pre
Post
No Early PD
Early PD
A.U.
HVEM
1200
4000
900
3000
600
2000
300
1000
0
0
Pre
Post
Pre
Post
No Early PD
Early PD
CD152
1000
300
750
250
500
200
150
250
100
0
Pre
Post
Pre
Post
No Early PD
Early PD
CD27
20000
12000
15000
10000
8000
5000
4000
0
Pre
Post
Pre
Post
No Early PD
Early PD
A.U.
IDO
300
300
200
200
100
100
0
Pre
Post
Pre
Post
No Early PD
Early PD
BTLA
500
900
400
600
300
300
200
0
100
Pre
Post
Pre
Post
No Early PD
Early PD
LAG3
150
60
100
40
50
20
0
Pre
Post
Pre
Post
No Early PD
Early PD
A.U.
PD-1
25
150
20
100
15
50
10
5
0
Pre
Post
Pre
Post
No Early PD
Early PD
CD80
40
150
30
100
20
50
10
0
Pre
Post
Pre
Post
No Early PD
Early PD
GITR
40
20
20
10
0
0
Pre
Post
Pre
Post
No Early PD
Early PD
A.U.
PD-L1
100
2000
1500
75
1000
50
500
25
0
Pre
Post
Pre
Post
No Early PD
Early PD
PD-L2
150
80
100
60
50
40
0
Pre
Post
Pre
Post
No Early PD
Early PD
A.U.
Suppl. Fig. 6. Longitudinal analsiys of plasma soluble immune checkpoints in patients experiencing early PD vs non-early PD in cohort A. Soluble immune checkpoint dynamics in baseline vs 4 cycles of ICI therapy in early PD vs non early PD patients in Cohort A. The Wilcoxon paired test revealed no statistically significant differences between pre and post.

## Slide 7
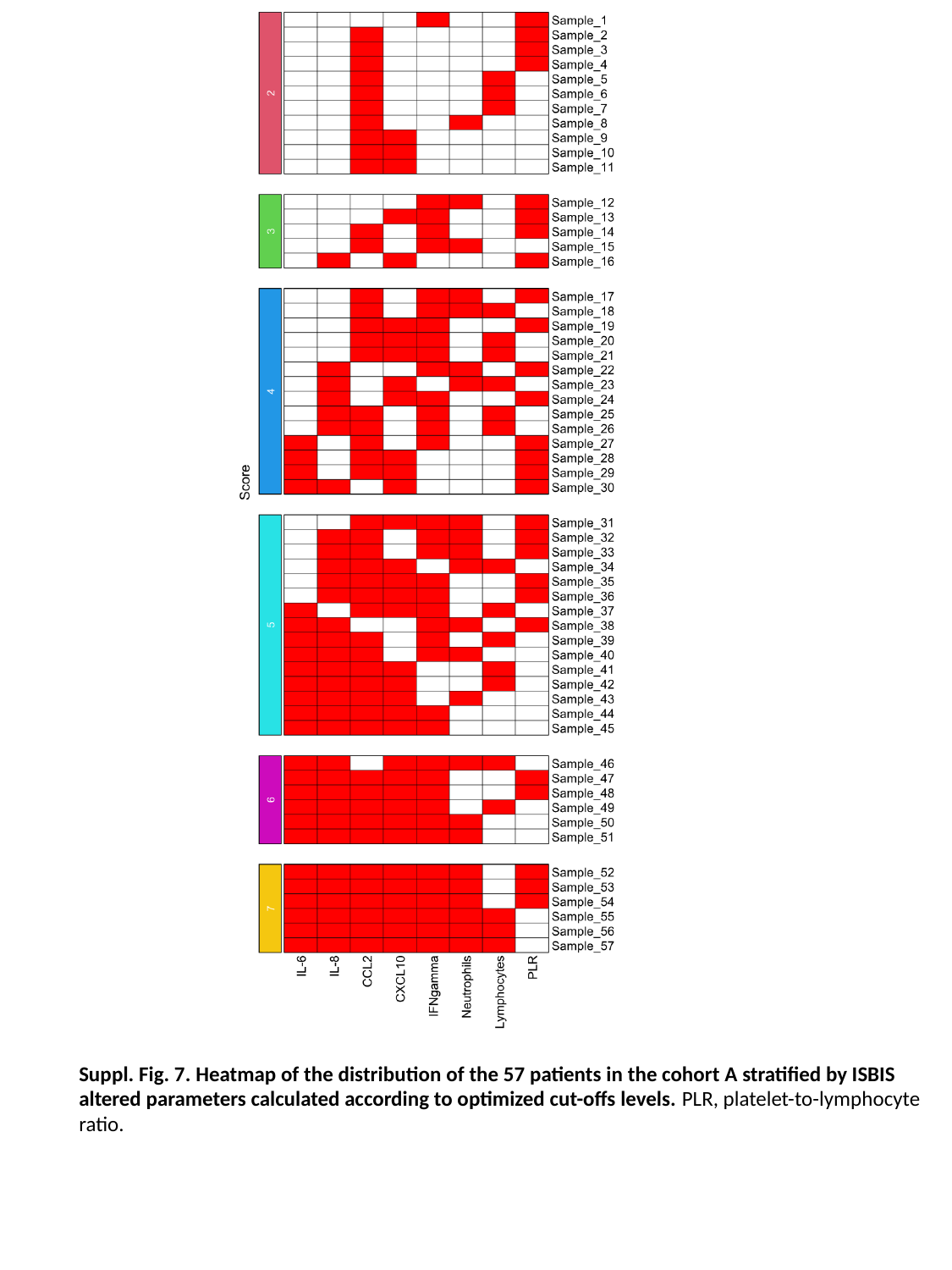

Suppl. Fig. 7. Heatmap of the distribution of the 57 patients in the cohort A stratified by ISBIS altered parameters calculated according to optimized cut-offs levels. PLR, platelet-to-lymphocyte ratio.

## Slide 8
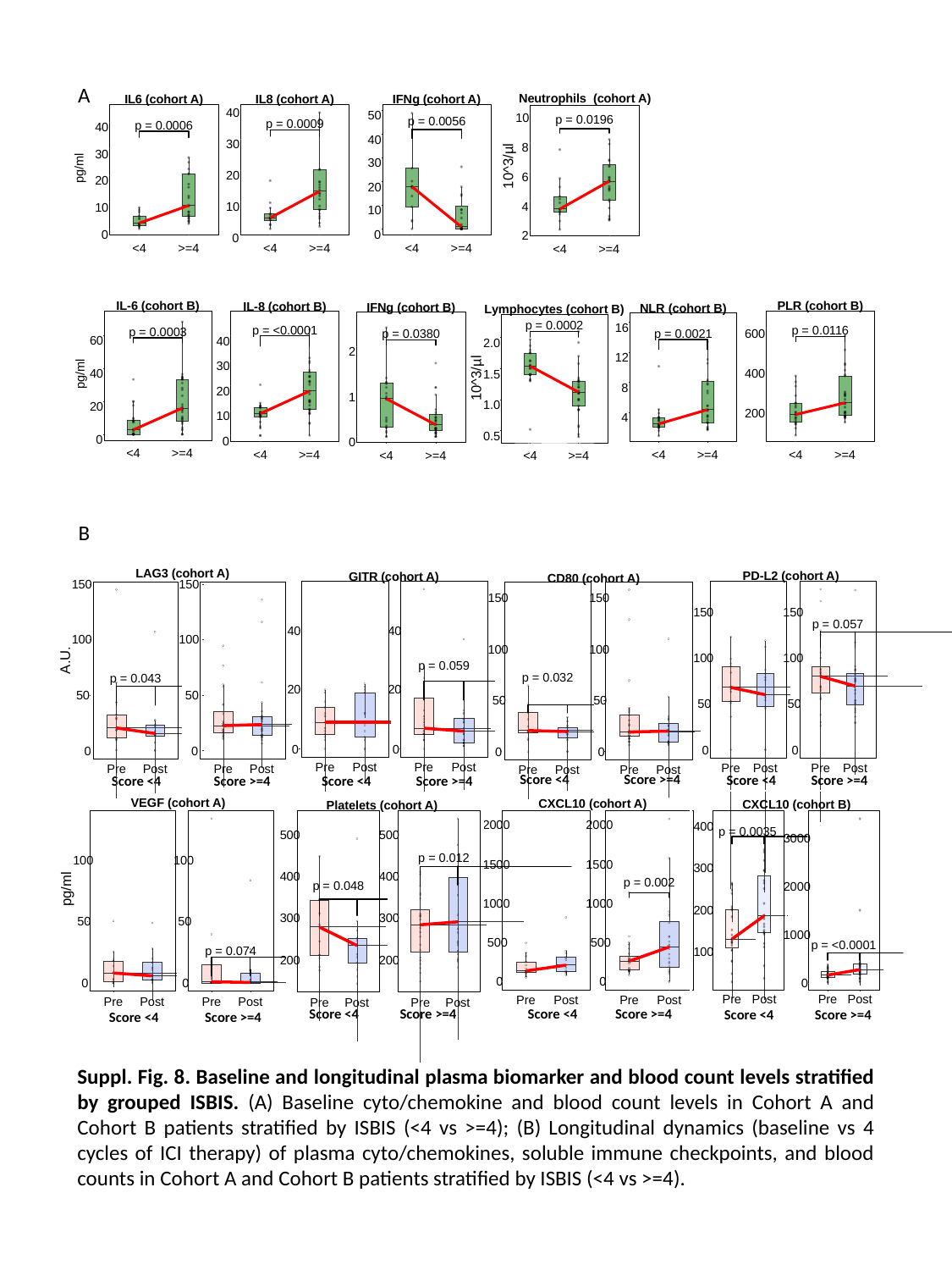

A
IL6 (cohort A)
40
p = 0.0006
30
20
10
0
<4
>=4
IL8 (cohort A)
40
p = 0.0009
30
20
10
0
<4
>=4
IFNg (cohort A)
50
p = 0.0056
40
30
20
10
0
<4
>=4
10
p = 0.0196
8
6
4
2
<4
>=4
Neutrophils (cohort A)
10^3/µl
pg/ml
PLR (cohort B)
600
p = 0.0116
400
200
<4
>=4
IL-6 (cohort B)
p = 0.0003
60
40
20
0
<4
>=4
IL-8 (cohort B)
p = <0.0001
40
30
20
10
0
<4
>=4
Lymphocytes (cohort B)
p = 0.0002
2.0
1.5
1.0
0.5
<4
>=4
NLR (cohort B)
16
p = 0.0021
12
8
4
<4
>=4
IFNg (cohort B)
p = 0.0380
2
1
0
<4
>=4
pg/ml
10^3/µl
B
LAG3 (cohort A)
150
150
100
100
p = 0.043
50
50
0
0
Pre
Post
Pre
Post
Score <4
Score >=4
PD-L2 (cohort A)
GITR (cohort A)
CD80 (cohort A)
150
150
150
150
p = 0.057
40
40
A.U.
100
100
100
100
p = 0.059
p = 0.032
20
20
50
50
50
50
0
0
0
0
0
0
Pre
Post
Pre
Post
Pre
Post
Pre
Post
Pre
Post
Pre
Post
Score <4
Score >=4
Score <4
Score >=4
Score <4
Score >=4
VEGF (cohort A)
CXCL10 (cohort A)
2000
2000
1500
1500
p = 0.002
1000
1000
500
500
0
0
Pre
Post
Pre
Post
CXCL10 (cohort B)
Platelets (cohort A)
400
p = 0.0035
500
500
3000
p = 0.012
100
100
300
pg/ml
400
400
p = 0.048
2000
200
300
300
50
50
1000
p = <0.0001
p = 0.074
100
200
200
0
0
0
Pre
Post
Pre
Post
Pre
Post
Pre
Post
Pre
Post
Pre
Post
Score <4
Score >=4
Score <4
Score >=4
Score <4
Score >=4
Score <4
Score >=4
Suppl. Fig. 8. Baseline and longitudinal plasma biomarker and blood count levels stratified by grouped ISBIS. (A) Baseline cyto/chemokine and blood count levels in Cohort A and Cohort B patients stratified by ISBIS (<4 vs >=4); (B) Longitudinal dynamics (baseline vs 4 cycles of ICI therapy) of plasma cyto/chemokines, soluble immune checkpoints, and blood counts in Cohort A and Cohort B patients stratified by ISBIS (<4 vs >=4).
